# Issues with incorrect computing of population attributable fraction (PAF) in a global perspective on coal-fired power plants and burden of lung cancer

**DOI:** 10.1186/s12940-019-0490-6

**Published:** 2019-06-13

**Authors:** Ahmad Khosravi, Mohammad Ali Mansournia

**Affiliations:** 1 Department of Epidemiology, School of Public Health, Shahroud University of Medical Sciences, Shahroud, Iran; 2 Department of Epidemiology and Biostatistics, Tehran University of Medical Sciences, Tehran, Iran

**Keywords:** Population attributable fraction, Confounding, ecological study

## Abstract

All observational studies are liable to confounding and Levin’s formula becomes useless in practice for unbiasedly estimating PAF. With respect to causal interpretation of PAF in public health setting, unbiased estimation of PAF requires several assumptions which are ignored in practice. We recommend using Miettinen PAF formula with careful consideration about possibility of bias in study design and analysis.

To the Editor

We read with great interest a recent article titled [1]: “A global perspective on coal-fired power plants and burden of lung cancer”. The authors evaluated the association of capacity of coal-fired power plants with lung cancer incidence at the national level using an ecological study design. In the burden of diseases analysis section it was indicated that population attributable fraction (PAF) of lung cancer to capacity of coal-fired power plants in 2015 was estimated using Levin’s formula [2]. In the study, authors quantified adjusted relative risks of lung cancer incidence given coal capacity at year t-10 for every country using RR_it_ = RR_0_^per capita coal capacity i(t-10)^. They calculated standardized attributable cases using *PAF*_*it*_.

1 $$ {PAF}_{it}=\frac{p_e\left({RR}_{it}-1\right)}{p_e\left({RR}_{it}-1\right)+1} $$

However, there are several concerns in the analysis:

1. There is a mistake in the calculation of PAF using proportion of males or females as p_e_. In the Levin’s formula, p_e_ is the proportion of the population exposed to the risk factor. The assumption of spatial homogeneity in exposure distribution in country level was implicit in the definition of risk factor i.e. per capital coal capacity; therefore p_e_ would be equal to 1. In this case Levin’s formula is equal to ((RR-1)/RR).
2. Levin’s formula is unbiased in the absence of confounding and effect modification [3, 4]. All observational studies are liable to confounding and formula 1 becomes useless in practice for unbiasedly estimating PAF [3]. The Meittinen formula (PAF = p_c_ (RR_adj_-1)/RR_adj_) is appropriate for use in practice as it provide unbiased estimate of PAF with adjusted RR when confounding exists [3]. Opposed to the Levin’s formula, it requires information about the prevalence of exposure among the cases (p_c_). As p_e_ is equal 1, implies p_c_ is equal one, the Levin and Meittinen formulas will give the same results in this special case.
3. One of the main assumptions underlying the PAF is no bias in the study design. In this observational, ecological study, data were collected for 83 countries and the unit of analysis was country. Thus, in an ecological study when the data were aggregated, the outcome measures are likely to be biased [5]. Ecological fallacy is another misinterpretation of ecological study results. Another issue is that RRs in PAF calculation were derived from sex-specific analysis, but all values of confounding variables except of smoking were not sex-specific. It is necessary to know that the ecological studies must use for generating hypothesis rather than deriving an adjusted association between risk factors and diseases.
4. The authors used a longitudinal Poisson model to analyze the association of per capita coal capacity with incidence rate of lung cancer. In this model the exponentiated coefficients are incidence-rate ratio not risk ratio. However, when the disease is uncommon, odds ratio (OR), rate ratio and hazard ratio (HR) can be used instead of RR in Menttinen formula [3].

In sum, unbiased estimation of PAF requires several assumptions which are ignored in practice. We recommend using Miettinen PAF formula with careful consideration about possibility of bias in study design and analysis [3].

Yours Sincerely,

Dr. Ahmad Khosravi.

Dr. Mohammad Ali Mansournia.

## Abbreviation

PAF: Population attributable fraction
